# High-Risk Fertility Behavior and Its Associated Factors Among Women in Wad Madani Maternity Hospital, Sudan: A Cross-Sectional Study

**DOI:** 10.3390/healthcare14183041

**Published:** 2026-09-16

**Authors:** Nagat B. Elhag, Albagir M. Hassan, Nadiah AlHabardi, Ishag Adam

**Affiliations:** 1 Wad Madani Maternity Hospital, Wad Medani College of Medical Sciences and Technology, Wad Medani 5118, Sudan; ashmana552@gmail.com; 2 Department of Obstetrics and Gynecology, Faculty of Medicine, Umm Al-Qura University, Mekkah 24381, Saudi Arabia; amahassan@uqu.edu.sa; 3 Department of Obstetrics and Gynecology, College of Medicine, Qassim University, Buraidah 51432, Saudi Arabia; ia.ahmed@qu.edu.sa

**Keywords:** pregnancy, high-risk, prevalence, education, age, body mass index, Sudan

## Abstract

**Background:** The literature indicates that high-risk fertility behavior (HRFB) is prevalent in resource-limited settings. However, there is currently no data available on this issue in Sudan. This study aimed to investigate the prevalence and factors associated with HRFB among Sudanese women at Wad Madani Maternity Hospital in Gezira State, Sudan. **Methods:** A hospital-based cross-sectional study was conducted between 1 September and 1 December 2023. A total of 380 pregnant women who gave birth at Wad Madani Maternity Hospital were enrolled. The women’s sociodemographic characteristics and obstetrical data were assessed using a face-to-face questionnaire. The primary outcome was HRFB. Primary associated factors included maternal body mass index (BMI) and education level, while secondary outcomes focused on the distribution of individual risk categories. Multivariable binary regression analysis was performed. **Results:** The median (interquartile range [IQR]) age and parity of the enrolled women were 26.5 (23.0–30.0) years and 2 (1–4), respectively. Of 380 women, 171 (45.0%, 95% confidence interval [CI] = 39.9–50.0%) had HRFB and 209 (55.0%, 95% CI = 49.9–60.1%) had no HRFB. Fifty-four (14.2%, 95% CI = 10.7–17.7%) women were classified as having multiple HRFBs, defined as the presence of two or more risk factors, and 117 (30.8%, 95% CI = 26.3–35.6%) had a single HRFB. In the multivariable binary regression analysis, high BMI (adjusted odds ratio [AOR] = 1.08, 95% CI, 1.03–1.13) and low maternal education level (AOR = 2.21, 95% CI, 1.45–3.37) were associated with having an HRFB. No significant associations were found between maternal employment and HRFB. **Conclusions:** The study revealed a high prevalence of HRFB (45.0%) among women in Wad Madani Maternity Hospital, Sudan, which was associated with low maternal education levels and BMI. Addressing these factors is needed to improve maternal and perinatal health outcomes in this hospital.

## 1. Introduction

High-risk fertility behavior (HRFB) refers to numerous factors that can make pregnancy high-risk or complicated. These behaviors increase the risk of mortality or immediate or delayed morbidity for the mother or her baby [1]. These factors include pregnancy at a very young age (<18 years) or advanced maternal age (>35 years), short inter-pregnancy intervals (IPIs) (<24 months), and high parity (≥4 births) [2]. HRFB is predominant within populations that suffer from economic crises, lack basic infrastructure, face healthcare shortages in terms of personnel or qualified health facilities, and face transportation issues and nutritional deficiencies [3], all of which make it even more challenging to meet the increased needs for optimal care during pregnancy and delivery.

The prevalence of HRFB is often high in developing countries. In Bangladesh, 46.0% of all births involve HRFB [4]; in India, the reported prevalence of HRFB is almost 30.0% [1]; in sub-Saharan Africa, it reaches 77.7% [5]; and in Ethiopia, HRFB was found in 67.3% of women in 2023 [6]. Similar results were found in Kenya, where HRFB was seen in 70.9% of women in a large multilevel study [7]. The high prevalence of HRFB in developing countries may be attributed to several factors, including low maternal education [1,7,8,9], low paternal education [7,8,9], residence [1,7,10], and inadequate antenatal care (ANC) [1,6]. A lack of maternal education is an important factor in these countries, as poor education might be associated with a lack of knowledge about sexual and reproductive health [5,10,11]. Early marriage is also a risk factor for HRFB, which might be due to the availability of contraceptive methods and higher parity [9,12]. The education level of husbands or partners is directly related to HRFB, with lower education levels associated with a higher prevalence of HRFB [8,9].

Women living in rural areas have more HRFB than those living in urban areas, which might be explained by a lack of education and difficulties accessing healthcare services, including contraceptive services [13,14]. The tradition of marriage at a younger age is strongly related to more HRFBs, as women who get pregnant at an age below 18 have a higher risk of adverse maternal and neonatal outcomes than those who are older [15]. Women with HRFB might have a single risk factor or multiple behavioral risk factors. Maternal mortality remains high among populations in which HRFB is prevalent, especially concerning pregnancy at a very young age <18 years) [1]. Short inter-pregnancy intervals (IPIs) predispose mothers to increased risks of anemia, perinatal mortality, and low birth weight [4,16]. High-order parity is also a significant risk factor for maternal mortality and is associated with maternal undernutrition [17].

HRFB is a significant health problem associated with adverse maternal, neonatal, and child outcomes and has a negative impact on health services, especially in underdeveloped countries [18]. Therefore, efforts are needed to reduce the prevalence of HRFB by raising awareness of its adverse outcomes and risk factors. For instance, a national effort to increase education, improve awareness of marriage at young ages, and increase the availability of contraceptive services to reduce high parity would improve health promotion among women with HRFB.

While several studies have been conducted in African countries and have shown a high prevalence rate of HRFB, no study has explored HRFB in Sudan. In this context, an investigation into the fertility behaviors of women giving birth in Sudan is required to provide valuable insights into the challenges they face and to inform targeted interventions. Identifying the prevalence and associated factors for HRFB can help healthcare providers and policymakers develop strategies to promote safer reproductive practices and improve maternal health outcomes in the region. Therefore, this study aimed to investigate the prevalence and factors associated with HRFB among Sudanese women at the Wad Madani Maternity Hospital in Gezira State, central Sudan.

## 2. Methods

### 2.1. Study Area

Gezira State is one of the 18 states in Sudan. The 2008 census estimated the total population of Gezira State to be around 4 million [19]. Gezira State is home to three distinct ethnic groups with vastly different demographic behaviors [20]. Wad Madani Maternity Hospital is a tertiary hospital located in the center of Wad Madani City, providing maternity services to all women in Gezira and the surrounding states.

### 2.2. Participants and Study Design

This hospital-based cross-sectional study was conducted between 1 September and 1 December 2023, and enrolled women who presented to the labor ward for delivery at Wad Madani Maternity Hospital. The guidelines provided by the Strengthening the Reporting of Observational Studies in Epidemiology (STROBE) initiative were strictly followed [21].

### 2.3. Inclusion and Exclusion Criteria

From that initial index, every subsequent fifth (see the details below) woman admitted to the labor and delivery unit was formally invited to participate in the study. To ensure a comprehensive assessment of HRFB in a tertiary setting, we included both spontaneous admissions and referred cases, as well as both singleton and multiple gestations. Eligible women had to meet specific inclusion criteria, including giving birth in the hospital, being able to provide informed consent, and being free from physical disabilities that would impede anthropometric assessments. Women were not excluded based on pregnancy complications (such as preeclampsia or gestational diabetes), as these conditions are often clinical sequelae of the high-risk fertility behaviors being studied. For women with multiple births, parity was recorded based on the most recent delivery to maintain consistency in parity data.

### 2.4. Sampling

A systematic random sampling technique was employed to select and enroll the required sample size of women for this study. To establish the sampling interval (*k*), we conducted a retrospective review of hospital delivery records from the three months immediately preceding the active study period (June to August 2023). This review identified a total historical volume of 1834 deliveries, which served as our baseline proxy for expected patient flow.

The sampling interval (*k*) was calculated by dividing the total expected population size (N = 1834) by the predetermined target sample size (n = 380). This calculation (1834/380 = 4.8) was rounded to the nearest whole integer, yielding an interval of (*k* = 5). To initiate the sequence without introducing selection bias, a random number between 1 and 5 was chosen as the starting point. If the selected woman did not fulfill the criteria, or did not agree to be enrolled, the next woman was enrolled, and a five-patient interval was maintained.

The recruitment process began in September 2023 and continued sequentially until the final enrollment threshold was satisfied. During the actual period of the study, 1906 women presented to the clinic, which was similar to the number of women (1834) who presented previously before starting the study. Data collection concluded in December 2023, with a final sample size of 380 women. Because this study used a cross-sectional design, all relevant clinical and demographic data were collected concurrently at enrollment. No longitudinal follow-up or subsequent contact was conducted with the participants after their initial assessment.

### 2.5. Sample Size Calculation

The sample size was calculated using a single population proportion formula using the OpenEpi Menu 3.01 (OpenEpi, Atlanta, GA, USA) [22]. A design effect of 1.0 was assumed, as the systematic sampling of daily hospital admissions in this single-center study was expected to approximate simple random sampling without the clustering effects typically seen in multi-stage community surveys. The authors estimated that 50% of women would have HRFB, given the current lack of data on this issue in Sudan. This assumption was based on the reported prevalence of HRFB in Ethiopia, which is 67.3% [6]. Additionally, based on the same study [6], the authors assumed that 50.0% of women with HRFB would have had inadequate ANC, while 32.0% of women without HRFB would have had adequate ANC. As a result, a sample size of 380 women was determined with a 95% confidence level and a difference of 5% at *α*  =  0.05, with a power of 80%. The authors also accounted for the assumption that 10% of participants would either not respond or have incomplete data.

### 2.6. Data Collection

Two trained (female) general practitioners, under the direct supervision of the research team, collected sociodemographic information and obstetrical data through face-to-face interviews using a questionnaire based on similar prior studies [1,6,7,8,9,10]. The questionnaire was adapted from validated tools, translated into Arabic, and back-translated to ensure conceptual accuracy. We conducted a pilot study on 10% of the sample to refine clarity and cultural relevance. Trained researchers administered the final Arabic version to ensure data quality and accommodate participants’ literacy levels. Information regarding maternal education and occupation was self-reported by the participants. To ensure the accuracy of reproductive data, the IPI and parity were verified by cross-referencing the participants’ maternal health cards and hospital records. IPI was defined as the time between the date that the previous pregnancy ended (live birth, stillbirth, miscarriage or abortion) and the date of the last menstrual period for the index pregnancy. To ensure data quality, the research supervisors reviewed all questionnaires daily for completeness and consistency. Because data were collected through direct face-to-face interviews and physical measurements, there was no missing data for enrolled participants.

### 2.7. Outcome Variable

Maternal HRFB was the outcome variable of this study. Per previous studies [6,7], HRFB was defined as the presence of any of four risks at participants’ last childbirth: maternal age less than 18 years or more than 35 years, parity equal or higher than 4, and a birth interval of less than 24 months. In this study, we used a modified definition: HRFB was defined as the presence of any of three risks at participants’ last childbirth: maternal age more than 35 years, parity equal or higher than 4, and an IPI of less than 24 months. The definition was modified because, currently, under Sudanese law, no certificate of marriage is issued to those under 18 years old. Thus, age was considered high risk if it was more than 35 years. Essential data, including maternal age, parity, and IPI, were collected from the mothers. The outcome variable was analyzed as dichotomous, coded as “0” if a woman did not report any high-risk fertility behaviors and “1” if she did. Additionally, for descriptive purposes, we categorized the intensity of risk: ‘single HRFB’ was defined as the presence of exactly one risk factor, while ‘multiple HRFB’ was defined as the simultaneous presence of two or more of the three risk factors (age > 35, parity ≥ 4, or IPI < 24 months). In this study, HRFB was assessed at the time of the current delivery. For multiparous women, risk factors included current age, total parity, and the IPI preceding the current birth. For primiparous women, assessment was limited to age-related risk. IPI was defined as the interval between the preceding live birth and the conception of the current pregnancy, verified, where possible, via medical records to reduce recall bias.

### 2.8. Explanatory Variables

Sociodemographic and reproductive factors associated with HRFB, as identified in previous studies, were included in the study. These factors included residence (urban or rural), body mass index (BMI), couple’s educational level, and mother’s occupation (housewife or employed). Couple’s educational level had two possible values: (1) less than secondary, i.e., participants who had no formal education or had only completed primary/intermediate schooling; and (2) secondary and above, i.e., participants who had completed secondary school (high school) or attained higher education degrees (university or postgraduate). ANC was considered adequate (≥8 visits) or inadequate (<8 visits). This threshold was adopted in accordance with the 2016 WHO ANC Model [23]. Participants’ weight and height were measured according to standard procedures at the time of admission to the labor ward, and BMI was computed using the formula weight (kg)/height (m^2^), and it was divided into underweight, normal weight, overweight, and obese according to the WHO classification [24]. As these measurements were taken during the third trimester, they reflect at-delivery BMI, including gestational weight gain.

### 2.9. Statistical Analysis

Data were analyzed using SPSS for Windows, version 25.0 (IBM Corp., Armonk, NY, USA). Continuous variables, such as age and BMI, were assessed for normality using the Shapiro–Wilk test and were found to be non-normally distributed. The results were expressed as proportions or medians with interquartile ranges (IQR). Initially, univariable binary logistic regression analysis was performed, with HRFB as the dependent variable and sociodemographic variables (such as education, residence, occupation, and BMI) and obstetrical variables (such as ANC) as independent variables. The mentioned independent variables were selected based on their associations in previous studies [1,6,7,10]. However, in this study, variables with a univariable *p*-value < 0.20 were considered for inclusion in the logistic regression analyses to adjust for potential confounders. This threshold was chosen to ensure that potential confounders were not prematurely excluded, allowing for a more robust adjustment in the final model. Before finalizing the model, diagnostic checks were performed to ensure statistical validity [25]. Multicollinearity among the independent variables was assessed using the Variance Inflation Factor (VIF); values > 4 were considered indicative of multicollinearity, and none were detected. The goodness -of-fit of the final logistic regression model was evaluated using the Hosmer–Lemeshow test, with a *p*-value > 0.05 indicating an adequate fit. Variables involved in the definition of HRFB, such as age, parity, and IPI, were not included in the multivariable logistic regression. Finally, at the multivariable level, binary logistic regression was used to identify the factors associated with HRFB among women. Adjusted odds ratios (AORs) and 95% confidence intervals (CIs) were calculated, and a *p*-value < 0.05 was considered statistically significant.

## 3. Results

### 3.1. General Characteristics

During the study period, 402 women were initially approached based on the systematic sampling interval. Of these, 12 were excluded because they did not meet the inclusion criteria (e.g., physical inability to undergo height/weight measurement), and 10 declined to participate. A final total of 380 women were enrolled and included in the analysis (response rate of 94.5%). The median (IQR) age and parity were 26.5 (23.0–30.0) years and 2 (1–4), respectively. The medians (IQRs) of BMI and IPI (number of women who had IPI = 258) were 25.7 (22.5–28.2) kg/m^2^ and 24.6 (18.0–36.0) months, respectively. Of 380, 325 women (85.5%) resided in urban areas; 187 (49.2%) of the women had a secondary education or higher; 205 (53.9%) of the husbands had a secondary education or higher; and 177 (46.6%) of the women were formally employed. A total of 201 (52.9%) women had adequate ANC, while 179 (47.1%) women had inadequate ANC. Of the total 380, 155 (40.8%), 17 (4.5%), 151 (39.7%), and 57 (15.0%) were normal weight, underweight, overweight, and obese, respectively (Table 1). In this study, BMI measured at delivery reflects both maternal adiposity and gestational weight gain and should not be interpreted as pre-pregnancy BMI.

### 3.2. Prevalence and Factors Associated with HRFB

Of 380 women, 171 (45.0%, 95% CI = 39.9–50.0%) had HRFB and 209 (55.0%; 95% CI = 49.9–60.1%) had no HRFB. Fifty-four (14.2%, 95% CI = 10.7–17.7%) women were classified as having multiple HRFBs, defined as the presence of two or more risk factors, and 117 (30.8%,95% CI = 26.3–35.6%) had a single HRFB. Of the total 258 women who delivered before, 113 (43.7%, 95% CI = 37.9–49.9%) had a short IPI (<24 months). Of 380 women, 25 (6.6%, 95%CI = 4.1–9.1%) were older age (>35 years), and 98 (25.8%, 95%CI = 21.4–30.2%) had a high parity (≥4). In the univariable binary regression analysis, BMI, maternal education, and husband’s education were associated with HRFB. Residence, employment, and ANC showed no significant associations (Table 2).

In the multivariable binary regression analysis, an 8% increase in the odds of HRFB per 1 kg/m^2^ increase in BMI (AOR = 1.08, 95 CI, 1.03–1.13). Low maternal education level (AOR = 2.21, 95% CI, 1.45–3.37) was associated with HRFB; maternal employment showed no association with HRFB (Table 3). The model had an adequate fit; the Hosmer–Lemeshow test *p*-value was 0.861, and it had modest explanatory power (Nagelkerke R^2^ was 0.105).

## 4. Discussion

This is the first study to address HRFB in Sudan. It revealed a high prevalence of HRFB (45.0%) among women at Wad Madani Maternity Hospital, Sudan, with significant associations found between HRFB, high BMI, and low maternal education levels. This is slightly lower than the high rates of HRFB reported in other African countries, such as Chad (89.9%) [5], Ethiopia (67.3%) [6], and Kenya (70.8%) [7]. In contrast, low HRFB prevalence rates have been reported in South Africa (34.1%) [5] and India (30.0%) [1]. The high prevalence of HRFB in Sudan compared with other countries can be attributed to several sociocultural and economic factors, particularly given the defined criteria for HRFB: maternal age (>35 years), parity (fourth or higher), and short IPI.

Teenage pregnancy has previously been recognized as an important health concern in Sudan [26]. Many women become mothers before the age of 18, leading to a higher prevalence of HRFB [26]. As mentioned above, the issue of early marriage was governed by the law (it can be violated in rural areas). Other countries often have more stringent social or legal frameworks that delay the childbearing age [27].

The value placed on large families in Sudan can contribute to women having four or more children, which increases the risk of complications associated with high parity [28]. In countries with more access to family planning services and education, women tend to have fewer children [29].

Limited access to contraceptive methods and family planning education can result in shorter IPIs (<24 months) in Sudan [30]. This can lead to increased maternal and infant health risks [31]. In other countries, where family planning is more widely practiced and accepted, longer birth intervals are common. Recently, several studies from various countries, including our research in Sudan and systematic reviews, have reported that both short and long IPIs are associated with maternal and perinatal outcomes [32,33]. This emerging evidence calls for a redefinition of HRFB, particularly regarding the fertility risks associated with IPIs of less than 24 months.

High levels of poverty, limited access to education, and inadequate healthcare services in Sudan exacerbate HRFB. Furthermore, the ongoing conflict has detrimental effects on both health and the economy [34]. These factors hinder women’s ability to make informed reproductive choices. Countries with better economic conditions and healthcare infrastructure typically have lower rates of HRFB [5]. Overall, the combined effects of cultural practices, economic challenges, and limited access to education and healthcare contribute to the high prevalence of HRFB in Sudan compared to other countries. Addressing these issues through targeted interventions and education can help reduce HRFB and improve maternal and perinatal health outcomes.

This study revealed that HRFB was associated with low maternal education in Sudan. This finding aligns with other studies, particularly those from African countries [1,7,9,10]. For example, in Kenya, Seifu et al.’s study of 15,483 women of reproductive age showed that primary, secondary, and higher educational levels were associated with HRFB [7]. Several interrelated factors can explain the association between HRFB and low maternal education in Sudan. Women with low education levels often lack awareness of reproductive health issues, family planning options, and the potential risks associated with early childbearing and short birth intervals [26,31]. Additionally, limited education can restrict women’s access to healthcare resources and support, reducing their ability to make informed decisions about their reproductive health [35]. Cultural norms may perpetuate the cycle of low education and high fertility, highlighting the need for targeted educational interventions to improve maternal health outcomes in the region.

This study showed that a high BMI was associated with HRFB. In Bangladesh, Howlader et al. found that overweight/obesity was positively associated with HRFB in univariate analyses [36]. Our previous study in central Sudan reported that maternal obesity was positively associated with increasing age [37]. Moreover, a previous study in central Sudan reported that parity was higher among women with a higher BMI and who were overweight or obese [38]. Limited access to education and family planning resources often exacerbates this issue, as women with high BMI may have less awareness or support regarding their reproductive health. The BMI measured at delivery in this study reflects both maternal adiposity and gestational weight gain and should not be interpreted as pre-pregnancy BMI.

This study found that maternal occupation, husband’s education, residence, or inadequate ANC were not associated with HRFB. While this is in line with another study’s findings that HRFB is not associated with the husband’s education [6], other studies have found that HRFB is associated with low husband education [7,9], residence [1,7,10], and inadequate ANC [1,6]. The lack of significant association between HRFB and ANC in this study can be explained by the usage of a new definition of adequate ANC (eight contacts or more), which is commonly used now [23], compared to other studies using the old definition (four contacts or more) [1,6]. Also, the lack of association between maternal occupation and ANC adequacy and high-risk fertility behavior should be interpreted with caution; the lack of statistical significance may stem from insufficient power to detect smaller effect sizes within certain occupational categories or from misclassification bias in the quality of ANC. It remains possible that while the number of ANC contacts was recorded, the content and counseling provided during those contacts varied, potentially masking their true influence on fertility choices.

Such discrepancies in the prevalence rates and associated factors of HRFB across studies should encourage researchers to investigate HRFB in their own countries or regions to inform the development of comprehensive localized solutions.

It seems that HRFB is primarily driven by a combination of socioecological, evolutionary, cognitive, and psychosocial mechanisms. Rather than only conscious recklessness, these behaviors stem from deeply ingrained mental processes, social pressures, and environmental adaptations [39].

### 4.1. Strengths and Implications of the Study

To the best of the authors’ knowledge, this is the first study to investigate HRFB in Sudan. This research has several strengths and implications that local authorities can use to improve maternal and perinatal health in the country. The strengths of the study include the following: it specifically targeted pregnant women who delivered at Wad Madani Maternity Hospital, providing valuable insights into HRFB within a defined group that can inform local healthcare practices and policies. Using multivariable logistic regression to identify associations between HRFB and factors (such as BMI and education level) enhances the robustness of the findings and allows for a nuanced understanding of the contributing factors. The study addresses a critical public health issue in central Sudan, highlighting the need for targeted interventions and policies to improve maternal and perinatal health outcomes and reduce HRFB in the region.

The study’s implications include the following: The findings can inform local health authorities and policymakers in developing targeted educational programs and interventions to improve maternal education (e.g., on issues related to early marriage) and promote healthy weight management as strategies to reduce HRFB. The study emphasizes the need to train healthcare providers to recognize and address HRFB, ensuring they can offer appropriate counseling and resources to pregnant women and their families. The results underscore the necessity for further research, including longitudinal studies, to better understand the causal relationships between HRFB and its associated factors, ultimately aiding in the development of effective public health strategies.

### 4.2. Limitations of the Study

This study has some limitations that should be acknowledged to enhance future research. As a cross-sectional study, this research captures data at a single point in time, making it difficult to establish causal relationships between HRFB and associated factors. Additionally, the hospital-based design and systematic sampling may introduce selection and periodicity biases, as facility-born participants might not fully represent the general population. This limits the generalizability of the findings to other healthcare settings and communities with different cultural, socioeconomic, and healthcare contexts. Other studies have reported different prevalence rates of HRFB between countries/regions [1,11]. Excluding maternal age <18 years from the conventional HRFB definition on the basis of Sudanese marriage law may underestimate HRFB and limit comparability with international studies. This is considered a methodological limitation, particularly because teenage pregnancy may still occur despite legal restrictions.

BMI was measured at delivery rather than pre-pregnancy. While high at-delivery BMI is associated with HRFB in our analysis, this measurement includes gestational weight gain and may not perfectly reflect the mothers’ pre-conception BMI status. The primary limitation of this paper is its failure to account for ethnicity, which is essential, as Gezira State is home to three distinct ethnic groups with markedly different demographic behaviors [20]. Failing to control for these distinct ethnic variables likely introduces significant noise into the data, such as the unexpectedly weak link between HRFB and low maternal education. Incorporating ethnicity into future studies will be vital for designing highly targeted healthcare interventions to improve maternal and perinatal outcomes. Furthermore, the timing of this study, conducted during active conflict in Sudan, presents both a unique snapshot of maternal health in crisis and a significant limitation regarding generalizability. Security challenges likely restricted hospital access for those in more remote or contested areas, potentially introducing a selection bias toward women with better resources or more acute medical complications. Therefore, sizable longitudinal studies are necessary to better understand the dynamics and temporal associations involved and to overcome these limitations.

## 5. Conclusions

This study highlights a concerning prevalence of HRFB (45.0%) among women at Wad Madani Maternity Hospital, Sudan. Associations were identified between HRFB and low maternal education levels. There is a need for targeted interventions to improve maternal education. Additionally, improving access to adequate healthcare for women is needed to address these issues within the community and ensure better reproductive health for women in the region.

## Figures and Tables

**Table 1:** General characteristics of the studied women who gave birth at Wad Madani Maternity Hospital, Sudan (n = 380).

Variables		Median	Interquartile Range
Maternal age, years		26.5	23.0–30.0
Parity		2	1–4
Body mass index, kg/m^2^		25.7	22.5–28.2
Inter-pregnancy interval, months (number = 258)		24.6	18.0–36.0
		Frequency	Percentage
Residence	Urban	325	85.5
	Rural	55	14.5
Antenatal care	≥8 contacts	201	52.9
	<8 contacts	179	47.1
Maternal education status	≥Secondary	187	49.2
	<Secondary	193	50.8
Husband’s education status	≥Secondary	205	53.9
	<Secondary	175	46.1
Maternal employment status	Employed	177	46.6
	Housewife	203	53.4
Body mass Index, groups	Normal	155	40.8
	Underweight	17	4.5
	Overweight	151	39.7
	Obese	57	15.0
High-risk fertility behavior	No	209	55.0
	Yes	171	45.0

Note: Continuous variables are expressed as median (interquartile range [IQR]) due to non-normal distribution as confirmed by the Shapiro–Wilk test. Categorical variables are presented as frequencies and percentages.

**Table 2:** Univariable regression of factors associated with high-risk fertility behavior (HRFB) for the studied women who gave birth at Wad Madani Maternity Hospital, Sudan (n = 380).

Variables		High-Risk Fertility Behavior (HRFB)		Univariable Analysis		*p*-Value
		Yes (n = 171)	No (n = 209)	Odds Ratio	95 CI	
		Median (Interquartile Range)				
Body mass index (weight [kg]/height [m]^2^)		29.0 (26.0–34.0)	25.0 (21.0–28.0)	1.08	1.03–1.13	<0.001
		Frequency	Percentage			
Residence	Urban	143 (83.6)	182 (87.1)	Reference		
	Rural	28 (16.4)	27 (12.9)	1.32	0.74–2.33	0.342
Antenatal care	≥8 contacts	89 (52.0)	112 (53.6)	Reference		
	<8 contacts	82 (48.0)	97 (46.4)	1.06	0.71–1.59	0.765
Maternal education status	≥Secondary	65 (38.0)	122 (58.4)	Reference		
	<Secondary	106 (62.0)	87 (41.6)	2.28	1.51–3.45	<0.001
Husband’s education status	≥Secondary	81 (47.4)	124 (59.3)	Reference		
	<Secondary	90 (52.6)	85 (47.7)	1.62	1.07–2.43	0.020
Maternal employment status	Housewife	82 (48.0)	121 (57.9)	Reference		
	Employed	89 (52.0)	88 (42.1)	1.49	0.99–2.34	0.054

**Table 3:** Multivariable logistic regression of factors associated with high-risk fertility behavior among women who gave birth at Wad Madani Maternity Hospital, Sudan (n = 380).

Variables		Adjusted Odds Ratio	95% Confidence Interval	*p*-Value
Body mass index, kg/m^2^		1.08	1.03–1.13	0.001
Maternal education status	≥Secondary	Reference		
	<Secondary	2.21	1.45–3.37	<0.001
Maternal employment status	Employed	Reference		
	Housewife	1.46	0.95–2.22	0.079
Husband’s education status	≥Secondary	Reference		
	<Secondary	1.50	0.93–2.41	0.089

Note: Odds Ratios (ORs) for body mass index represent the change in risk per 1 kg/m^2^ increase.

## Data Availability

The data supporting the current study’s findings are available from the corresponding author upon reasonable request.
